# Caregiver recruitment strategies for interventions designed to optimize transitions from hospital to home: lessons from a randomized trial

**DOI:** 10.1186/s13063-024-08288-2

**Published:** 2024-07-04

**Authors:** Allison M. Gustavson, Molly J. Horstman, Jodie A. Cogswell, Diane E. Holland, Catherine E. Vanderboom, Jay Mandrekar, William S. Harmsen, Brystana G. Kaufman, Cory Ingram, Joan M. Griffin

**Affiliations:** 1 Veterans Affairs Health Services Research and Development Center for Care Delivery and Outcomes Research, Minneapolis Veterans Affairs Health Care System, Minneapolis, MN 55417 USA; 2 Department of Medicine, University of Minnesota, Minneapolis, MN 55455 USA; 3 Center for Innovations in Quality, Effectiveness, and Safety, Michael E. DeBakey VA Medical Center, Houston, TX 77030 USA; 4 Department of Medicine, Baylor College of Medicine, Houston, TX 77030 USA; 5 Robert D. and Patricia E. Kern Center for the Science of Health Care Delivery Research, Rochester, MN 55905 USA; 6 Department of Quantitative Health Sciences, Mayo Clinic, Rochester, MN 55905 USA; 7 Department of Population Health Sciences, Duke University, Durham, NC USA; 8 Duke-Margolis Institute for Health Policy, Duke University, Durham, NC USA; 9 Center of Innovation to Accelerate Discovery and Practice Transformation, Durham VA Health Care System, Durham, NC USA; 10 Department of Community Internal Medicine, Geriatrics, and Palliative Care, Mayo Clinic, Rochester, MN 55905 USA; 11 Division of Health Care Delivery Research (HCDR), Mayo Clinic, Rochester, MN 55905 USA

**Keywords:** Caregiver, Recruitment, Clinical trial, Methods, Palliative care, Post-acute care

## Abstract

Challenges to recruitment of family caregivers exist and are amplified when consent must occur in the context of chaotic healthcare circumstances, such as the transition from hospital to home. The onset of the COVID-19 pandemic during our randomized controlled trial provided an opportunity for a natural experiment exploring and examining different consent processes for caregiver recruitment. The purpose of this publication is to describe different recruitment processes (in-person versus virtual) and compare diversity in recruitment rates in the context of a care recipient’s hospitalization. We found rates of family caregiver recruitment for in-person versus virtual were 28% and 23%, respectively (*p* = 0.01). Differences existed across groups with family caregivers recruited virtually being more likely to be younger, white, have greater than high school education, and not be a spouse or significant other to the care recipient, such as a child. Future work is still needed to identify the modality and timing of family caregiver recruitment to maximize rates and enhance the representativeness of the population for equitable impact.

## Introduction

The increasing recognition and value of family caregivers (FCGs) during the vulnerable transition from hospital to home has positioned FCGs as essential stakeholders in research. The dynamic and uncertain context surrounding transitions of care from hospital to home often results in new tasks placed on the FCG. Capturing the FCG experience, voice, and perception is critical to designing, implementing, and scaling effective interventions that promote positive outcomes at patient, caregiver, clinician, and system levels.

Intervening to support FCGs is a potential solution to stabilizing transition plans and reducing caregiver burden and stress. However, challenges exist to recruiting and retaining caregivers in research [1–11]. Patient electronic health records (EHRs) are often used to identify potential research participants. However, EHRs rarely include the FCG’s name, relationship to patient, and contact information, nor do they include the type and level of caregiving support needed [12]. Screening patient EHR data for patient characteristics that are associated with high caregiver need is another option; [10] however this process is time and resource intensive, precluding its use for larger studies. Traditional recruitment methods first identify potential participants, mail opt-out letters, and then wait 10 days before initiating contact. This approach may not be ideal for studies on care transitions because the compressed hospitalization time does not provide flexibility for contacting FCGs or allow extra time for FCGs to weigh decisions about participation. The first 1–2 weeks at home pose the highest risk for adverse events (e.g., rehospitalization, falls) [13–17]. Therefore, recruiting after hospitalization is not ideal for examining the effects of interventions designed to capture and facilitate the transition from hospital to home. These recruitment challenges further complicate efforts to recruit diverse FCGs (considering a mix of age, sex, ethnicity, etc.), threatening the internal and external validity of FCG interventions.

With the COVID-19 pandemic, we experienced additional recruitment challenges firsthand while conducting the technology-enhanced Transitional Palliative Care for Family Caregivers trial (TPC), a study evaluating the effect of a novel video intervention designed to support rural FCGs caring for palliative care patients during the transition from hospital to home. The purpose of this publication is to describe our different recruitment processes (in-person versus virtual) and compare diversity in recruitment rates between processes in the context of a care transition from hospital to home.

## Methods

### Overview of TPC intervention and trial

The TPC trial protocol is outlined in detail elsewhere [17] (trial registration: NCT03339271, November 8, 2017). Briefly, the purpose of the randomized controlled trial was to test the efficacy and cost-effectiveness of a video-based, nurse-led intervention in improving transitions for critically ill patients with life-limiting illnesses by targeting FCG health and well-being. The intervention involved teaching, guiding, and counseling FCGs to enhance caregiving knowledge and skills, while also meeting the FCG’s own health needs. Participants were FGCs living in rural areas who were recruited while the patient (hereafter referred to as care recipient) was hospitalized between 2018 and 2022 [17]. FCGs were randomly assigned to an attention control condition or the intervention. The attention control group received monthly phone calls from a team member to collect cost data. This approach was utilized to reduce attrition and account for nonspecific effects of the intervention that may occur due to any interaction with the research team [17]. The intervention began while the care recipient was hospitalized and continued for 8 weeks after hospital discharge. The Mayo Clinic Institutional Review Board (IRB) approved this study (# 17–005188). We used the CONSORT checklist when writing our report [18].

### TPC caregiver recruitment strategies

FCGs were recruited from four hospitals in the same health system in the upper Midwest. FCGs were broadly defined as persons who self-identified as an unpaid, informal caregiver for someone with unmet medical or care needs. Recruitment was timed to occur after a palliative care consult but prior to patient discharge from the hospital. The COVID-19 pandemic prompted a shift in recruitment strategies from in-person to virtual. In-person recruitment included a face-to-face interaction with paper consent, whereas virtual recruitment was conducted via telephone with electronic consent.

To recruit for the trial, we screened the EHR Palliative Medicine calendar daily to identify care recipients admitted for inpatient services and who received a Palliative Medicine consult, but had not yet been discharged from the hospital. Every care recipient was initially screened via chart review in the EHR to determine eligibility and identify if a FCG was named in the EHR. Care recipients, and thereby their FCGs, were excluded if they were < 21 years, had a left ventricular assist device, used home infusion pain pumps, or had documented chronic pain or addictive behaviors in their problem list. Following the initial eligibility screen of care recipients, FCGs were contacted, and the FCG eligibility was confirmed. To be included, FCGs had to provide care outside of the hospital, be ≥ 21 years old, and live in a rural or medically underserved setting (population of 50,000 and under) in Minnesota, Wisconsin, or Iowa. FCGs interested in participating needed to consent prior to the care recipient’s discharge from the hospital.

Prior to the COVID-19 pandemic, in-person recruitment and consent at the hospital was standard. A study coordinator would call the FCG to confirm eligibility, provide a short description of the study, and ask if the FCG could meet with them in-person when the FCG visited the care recipient at the hospital. If the FCG agreed, the study coordinator would arrange a time to meet the FCG at the hospital, give them a study brochure, review the study details outlined in the brochure, answer questions, and then obtain written consent from the FCG. If the FCG wanted to first think about the study, the study coordinator would leave a recruitment packet containing a consent and study brochure with the FCG, follow up within 2 days by phone or in person, and then collect paper consent forms in-person at the hospital.

With the onset of the COVID-19 pandemic hospital policies on March 18, 2020, virtual recruitment became standard. With visitor restrictions imposed, we pivoted our recruitment strategy to avoid any in-person contact and had a 2-week hiatus from recruitment while we revised our processes. We telephoned potential FCG participants to confirm eligibility, but then continued with recruitment activities by phone. The virtual process included a verbal overview of study details—instead of a brochure, time to answer questions, and a verbal review of the consent document. If the FCG agreed to consent to study participation, the study coordinator would enter their information into a Participant Tracking System (Ptrax) and send a secure link by electronic email for an electronic consent form (e-consent). The study coordinator was available to guide a potential participant through the electronic signature process upon request. The FCG was enrolled in the study once the electronic signature was received.

### Data collection and analyses

All data were captured in the REDCap [19] database hosted at the Mayo Clinic. Recruitment and retention data was collected by the study coordinator. Demographic data was collected from baseline study surveys or the care recipient’s EHR. FCG burden was assessed electronically prior to care recipient discharge using the 15-item (7-point scale with − 3 to + 3 ratings) Bakas Caregiving Outcomes Scale-Revised (BCOS-R), with higher scores representing better FCG outcomes [20]. The ratings were recoded to 1–7 to determine a positive value that would be used in the analysis. The care recipient’s risk for mortality at the time of discharge was assessed using the Charlson Comorbidity Index, with scores derived from the International Classification of Diseases (ICD)-10 codes available in the care recipient’s EHR. Scores range from 0 to 39, with scores > 5 are considered to have high comorbidity [21]. Discrete baseline characteristics were compared between the two recruitment modalities using a chi-square or Fisher’s exact test, as appropriate. Ordered self-report health status was compared using a Wilcoxon rank sum test and continuous variables were compared using a two-sample* t*-test. The comparison of recruitment rates between modalities was made using a chi square test. The alpha-level was set at 0.05 for statistical significance. All analyses were conducted using SAS version 9.4 (SAS Inc., Cary, NC).

## Results

### TPC caregiver recruitment rates

**Fig. 1:**
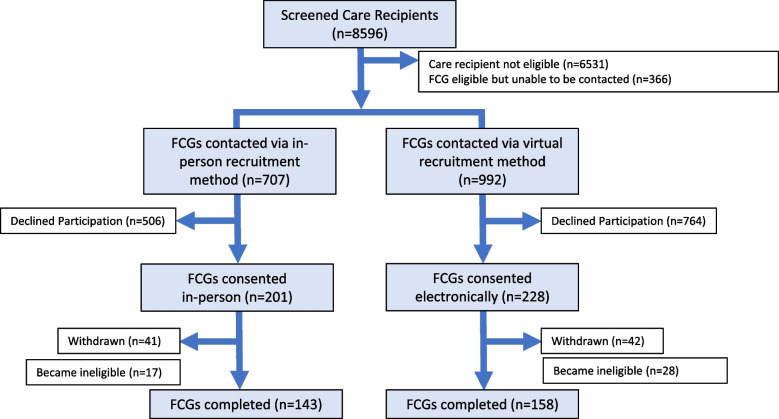
Participant flow diagram for TPC by method of recruitment

Figure 1 depicts the participant flow diagram by method of recruitment. Eight thousand five hundred ninety-six patients were screened and 1699 FCGs contacted (82% successful contact rate out of the 2065 eligible). Four hundred and twenty-nine FCGs (201 in-person; 228 virtual) provided consent to participate in the TPC trial. For in-person recruitment, 28% of FCGs contacted were recruited compared to 23% of FCGs contacted with virtual recruitment (*p* = 0.01). Twenty-three percent (*n* = 42) of FCGs recruited in-person later withdrew from the study compared to 21% (*n* = 41) of FCGs recruited virtually.

### TPC caregiver characteristics by recruitment modality

Table 1 presents FCG characteristics by recruitment modality. Compared to those recruited in-person, those recruited virtually were significantly younger (mean ± SD: 55.5 ± 11.8 vs. 61.4 ± 13.1 years; *p* < 0.0001) and more likely to be white (95.7% vs. 92.2%, *p* = 0.03). The group recruited virtually was significantly more likely to have vocational or college education (89.1% vs. 77.5%, *p* < 0.1) and work full-time (50.3% vs. 34.4%), yet they were less likely to be spouses (50.8% vs. 71.7%, *p* < 0.001) or live in the same home as the care recipient (65.7% vs. 82.1%, *p* < 0.001). More participants recruited virtually provided caregiving for others in addition to the care recipient, compared to the group recruited in-person (40.3% vs 24.2%; *p* = 0.004). However, fewer participants recruited virtually compared to in-person stated they received caregiving help from others (70.1% vs. 80.6%, *p* = 0.04). Characteristics of the care recipient (in-hospital mortality/medical complexity and—related—caregiver burden) were not significantly different between groups (*p* = 0.23). Participants did not differ significantly between recruitment methods by other factors possibly related to rates of recruitment such as self-reported health (*p* = 0.32). The mean duration from eligibility (receiving a palliative care consult) to hospital discharge and from the time of consent to the care recipient’s hospital discharge were not statistically different between groups (*p* > 0.26).

**Table 1:** Characteristics of participants and their care recipients by recruitment method

**Characteristic**	**Total** (*N* = 384)	**In-person recruitment** (*N* = 185)	**Virtual recruitment** (*N* = 199)	***P*-value**
	**Mean ± SD (*N*) or frequency (*N*)**			
Age (years), mean ± SD	58.3 ± 12.7 (349)	61.4 ± 13.1 (169)	55.5 ± 11.8 (180)	< 0.0001^1^
Sex Female Male	69.8% (268) 30.2% (116)	68.6% (127) 31.4% (58)	70.9% (141) 29.1% (58)	0.64^2^
Race White Non-White or ≥ 1 race	94.0% (297) 6.0% (19)	92.2% (142) 7.8% (12)	95.7% (155) 4.3% (7)	0.03^3^
Ethnicity Hispanic or Latino Not Hispanic or Latino	0.7% (2) 99.3% (283)	0.8% (1) 99.2% (130)	0.6% (1) 99.4% (153)	1.0^3^
Education < High school High school Vocational/college	1.4% (4) 14.6% (43) 84.0% (247)	3.1% (4) 9.4% (25) 77.5% (100)	0.0% (0) 10.9% (18) 89.1% (147)	< 0.01^3^
Employment Full-time (35 + h/week) Part-time (35 h/week) Leave of absence Not employed Retired	43.4% (126) 11.0% (32) 4.1% (12) 10.0% (29) 31.4% (91)	34.4% (43) 14.4% (18) 3.2% (4) 9.6% (12) 38.4% (48)	50.3% (83) 8.5% (14) 4.8% (8) 10.3% (17) 26.1% (43)	0.03^3^
Living in the same home as the care recipient No Yes	26.5% (101) 73.6% (281)	17.9% (33) 82.1% (151)	34.3% (68) 65.7% (130)	< 0.001^2^
Relation to care recipient Spouse/Significant other Parent Other	60.8% (233) 27.9% (107) 11.2% (43)	71.7% (132) 18.5% (34) 9.8% (18)	50.8% (101) 36.7% (73) 12.6% (25)	< 0.001^2^
Bakas Caregiving Outcome Scale Revised	4.5 ± 0.6 (294)	4.4 ± 0.6 (135)	4.5 ± 0.6 (159)	0.23^1^
Self-reported health status Poor Fair Good Very good Excellent	0.3% (1) 5.1% (15) 43.0% (126) 39.9% (117) 11.6% (34)	0.0% (0) 6.3% (8) 46.1% (59) 35.9% (46) 11.7% (15)	0.6% (1) 4.2% (7) 40.6% (67) 43.0% (71) 11.5% (19)	0.32^4^
Caregiving for additional person Yes, child Yes, older adult No	3.5% (10) 29.9% (86) 66.7% (192)	2.4% (3) 21.8% (27) 75.8% (94)	4.3% (7) 36.0% (59) 59.8% (98)	0.004^2^
Caregiving help from others Yes No	66.7% (192) 33.3% (96)	80.6% (100) 19.4% (24)	70.1% (115) 29.9% (49)	0.04^2^
Care Recipient Charlson Comorbidity Index	9.2 ± 3.8 (294)	9.1 ± 4.0 (135)	9.2 ± 3.7 (159)	0.83^1^
Days from caregiver consent to care recipient hospital discharge	6.7 ± 17.4 (384)	5.7 ± 8.2 (185)	7.6 ± 22.8 (199)	0.30^1^
Days from care recipient eligibility (palliative note in EHR) to hospital discharge	10.4 ± 19.3 (384)	9.3 ± 9.1 (185)	11.5 ± 25.4 (199)	0.26^1^

^1^Two-sample *T*-test

^2^Chi-square test

^3^Fisher’s exact test

^4^Wilcoxon rank sum test

## Discussion

We found rates of FCG recruitment higher for in-person compared to virtual recruitment for a randomized controlled trial conducted to test the effectiveness of an intervention to support FCGs during the transition of a critically ill care recipient from hospital to home. FCGs recruited virtually were more likely to be younger, white, have vocational or college level education and were less likely to live in the same residence as the care recipient, be spouses with the care recipient, provide care to another person in addition to the care recipient, and receive less caregiving help from others. This study adds to the literature by describing the reach and participant diversity when utilizing two FCG recruitment strategies in healthcare settings.

The modality in which recruitment and consent occur is an important contextual factor when recruiting FCGs. The wider variability in days from caregiver consent to care recipient hospital discharge and days from care recipient eligibility to hospital discharge in the virtually recruited group may have been an indicator of longer stays due to COVID-19 restrictions (e.g., lack of post-acute services, longer hospital stays). Alternatively, with the virtual recruitment process, we may have been able to connect with caregivers more quickly by not including and coordinating an in-person visit. A potential challenge to virtual recruitment and, subsequently, electronic consent is the reliance on the participant having access to email and the internet [22]. We found that internet connectivity was not a significant issue in this rural sample once recruited, but we are unsure if virtual methods introduced new biases due to virtual access. Age may play a role in receptivity to and enrollment of FCGs through virtual methods requiring internet/email and digital literacy [23, 24]. In the context of a hospitalization and our trial, we note that the differences in recruitment rates by approach may be due to limitations on when we could recruit and when caregivers might be available. For example, FCGs who were younger may not have been available pre-pandemic during business hours, whereas an older FCG may have been more available at bedside for in-person recruitment. These observations and perspectives support the need for multi-modal recruitment strategies that meet the needs, preferences, and capabilities of FCGs to participate in recruitment processes for caregiving studies. In our case, the COVID-19 pandemic provided a natural experiment to observe changes in recruitment rates when switching—by necessity—from in-person to virtual processes. In-person recruitment is a resource-intensive approach that requires travel time on the part of participants and research personnel. However, in-person does offer the opportunity for rapport [22]. Conversely, virtual recruitment and electronic consent allow flexibility in time and place for both researchers and potential participants. While the difference in recruitment rates between approaches was statistically different, there is a balance to be struck between the costs associated with in-person recruitment and the potential to contact more FCGs with virtual recruitment, but with a higher refusal rate. Research is needed to compare costs in recruitment strategies to identify the ideal return on investment in caregiver studies.

Our challenges with recruitment are shared with others across multiple settings and patient populations with prevailing issues in FCG identification, low recruitment rates, and high numbers of withdrawals from participation [3, 5, 7–9, 25, 26]. Opt-out recruitment approaches are common in healthcare delivery settings because of the ability to harness the EHR to screen for enrollment and identify contact information of potential FCGs [10]. Ma and colleagues employed an EHR-driven process to identify unpaid FCGs of Veterans and found that of the 2134 Veterans who received opt-out letters and were called, 64% answered, and—of those—60% had an unpaid FCG [10]. However, opt-out letters—while a common and successful strategy [3, 10] —are not feasible in the setting of a brief hospital stay. Provider outreach is possible, but likely yields low recruitment due to time constraints on the provider’s part, and difficulty integrating both outreach and communication to the research team into clinical workflow [3, 27].

Finally, we shared similar challenges to other researchers in reaching and recruiting racially or ethnically marginalized populations [3, 5, 7–9, 25]. Although more FCG participants in the in-person group compared to the virtual group represented greater racial diversity, most of the overall sample were white. The small proportion of Black, Asian, and Hispanic participants limited our power to detect differences in recruitment across racial groups. In addition, age may explain the other differences observed between FCGs by recruitment method, such as employment status, relationship to care recipient, self-reported health, and caregiving for an additional person. However, as an ancillary study, we are not powered to look at these comparisons. The modality and timing of recruitment likely play important roles in who is approached, consented, and enrolled. Virtual recruitment may reduce implicit bias that can hinder diversity in trial enrollment [28]. Importantly, we recruited from a health care system which has the advantage of using EHR data to assist in recruitment identification, but may bias participants who seek and have access to the healthcare system. Structural racism and mistrust in the healthcare systems may lead to missed opportunities to recruit a diverse pool of participants that is necessary to identify culturally sensitive adaptations to the intervention and intervention delivery [29–31].

Our trial spanned the early pandemic in 2020 to 2023 and, thus, our recruitment rates likely varied between the onset of the pandemic and the later stages as the world attempted to return to normal. Beyond the modality of recruitment, the pandemic likely changed people’s perceptions about participation in clinical trials; [32] as an ancillary study, our data is unable to discern between changes in recruitment due to modality and changes due to the experiences during the pandemic. The COVID-19 pandemic unmasked the persistent challenges in recruiting a diverse sample, regardless of in-person or virtual approaches. There is an opportunity to learn from the use of two different methods in a single trial to identify inequities and advocate for a multi-modal approach to recruitment. Further research is needed to better understand impacts of recruitment methods on equitable enrollment.

## Conclusions and lessons learned

Health care systems continue to recognize the essential role of FCGs in the health and well-being of care recipients and the potential health risks posed to the FCG through this role [6, 33–35]. However, challenges to FCG recruitment in the healthcare delivery setting exist and detrimentally impact the advancement of research in this area. To feasibly recruit FCGs during a hospitalization period, we found that utilizing multiple approaches to obtain consent can be a solution to optimizing recruitment and diversity in caregiving research. However, this may depend on the resources available within the research study. Future work is needed to identify the modality, timing, and cost-effectiveness of FCG recruitment to maximize recruitment rates and enhance the representativeness of the population for equitable impact. A qualitative study may be helpful prior to trial commencement with the caveat that this is a challenging population to recruit for any study. Post-intervention interviews with participants may yield insight into barriers as well as co-production of solutions to recruiting caregivers in the context of acute and post-acute care. Ideally, a multi-modal approach to recruitment would occur with the added flexibility of research staff to recruit outside of business hours. The hospitalization timeline is unpredictable, which garners the need for flexibility and adaptable approaches to fit the needs and capacity of researchers and FCGs alike.

## Data Availability

Data will be made available on reasonable request.
